# IgE Mediates Killing of Intracellular *Toxoplasma gondii* by Human Macrophages through CD23-Dependent, Interleukin-10 Sensitive Pathway

**DOI:** 10.1371/journal.pone.0018289

**Published:** 2011-04-22

**Authors:** Ioannis Vouldoukis, Dominique Mazier, Daniel Moynet, Denis Thiolat, Denis Malvy, M. Djavad Mossalayi

**Affiliations:** 1 Inserm U511, Université Pierre et Marie Curie, Paris, France; 2 Inserm U1035, UFR Sciences Pharmaceutiques, Université de Bordeaux, Bordeaux, France; City of Hope National Medical Center and Beckman Research Institute, United States of America

## Abstract

**Background:**

In addition to helminthic infections, elevated serum IgE levels were observed in many protozoal infections, while their contribution during immune response to these pathogens remained unclear. As IgE/antigen immune complexes (IgE-IC) bind to human cells through FcεRI or FcεRII/CD23 surface molecules, the present study aimed to identify which functional receptor may be involved in IgE-IC interaction with human macrophages, the major effector cell during parasite infection.

**Methodology/Principal Findings:**

Human monocyte-derived macrophages were infected with *Toxoplasma gondii* before being incubated with IgE-IC. IgE receptors were then identified using appropriate blocking antibodies. The activation of cells and parasiticidal activity were evaluated by mediator quantification and direct counting of infected macrophages. RNAs were extracted and cell supernatants were also collected for their content in tumor necrosis factor (TNF)-α, interleukin-10 (IL-10) and nitrites. Sera from symptomatic infected patients were also tested for their content of IgE, IL-10 and nitrites, and compared to values found in healthy donors. Results showed that IgE-IC induced intracellular elimination of parasites by human macrophages. IgE-mediated effect was FcεRI-independent, but required cross-linking of surface FcεRII/CD23, cell activation and the generation of nitric oxide (NO). Although TNF-α was shown to be produced during cell activation, this cytokine had minor contribution in this phenomenon while endogenous and exogenous IL-10 down-regulated parasite killing. Inverse relationship was found between IL-10 and NO expression by infected human macrophages at both mRNA and mediator levels. The relationship between these *in vitro* data and *in vivo* levels of various factors in *T. gondii* infected patients supports the involvement of CD23 antigen and IL-10 expression in disease control.

**Conclusion:**

Thus, IgE may be considered as immune mediator during antiprotozoal activity of human macrophages through its ability to trigger CD23 signaling. Increased cell activation by IgE-IC may also account for chronic inflammatory diseases observed in some patients.

## Introduction

Beside its critical role in allergy, IgE is generally believed to play a physiological role in immunity towards helminthic parasites [1]. Thus, *in vivo* expression of IgE has been observed during protozoal infections such as those caused by *Plasmodium spp.* [2], [3], *Leishmania spp.* [4] and *Trypanosoma cruzi* [5], although the role of this immunoglobulin in anti-microbial immunity remains unclear [6]. IgE/antigen bound to human cells through FcεRI and FcεRII surface molecules [7]. Macrophages, which are pivotal effectors for control of intracellular and extracellular parasites, fail to express FcεRI but may bound IgE through surface FcεRII/CD23 antigen [8]–[10], [11]. CD23 is distinguished structurally from almost all other immunoglobulin receptors as it belongs to the C-type (calcium-dependent) lectin superfamily [7]. It has been previously identified as a low affinity receptor for IgE on the surface of B lymphocytes, monocytes, follicular dendritic cells, Langerhans cells, eosinophils, epithelial cells and platelets. CD23 exhibits two isoforms, namely CD23a and CD23b, which are differentially expressed. CD23a is expressed by antigen-activated B cells before differentiation into antibody-secreting plasma cells, whereas CD23b expression by macrophages, B cells and a variety of inflammatory cells including epithelial cells is induced during immune response [7]. Moreover, expression of multimeric CD23b on the surface of various immune cells dramatically increased its ligand affinity [7], [12]. Consequently, CD23 plays a critical role during immune response including IgE synthesis, B- and T-cell differentiation, and the secretion of inflammatory mediators by various human cells [7]. Cross-linking of surface CD23 promotes the generation of IL-1, IL-6, TNF-α, H_2_O_2_ and iNOS-mediated NO through NFκB- and AP-1-dependent mechanisms [8], [13], [14]. As both IFN-γ and IL-4 promote surface CD23b expression in human macrophages, we and others demonstrated CD23 implication during both Th1 and Th2 immune responses [7], [8], [13]. In addition, soluble CD23 fragments, detected in human sera, mediate cell activation through the ligation of surface CD11b/c on macrophages [15], or CD21 on lymphocytes [16]. The role of CD23 during intracellular killing of intracellular *Leishmania* parasites [8] and mycobacteria [10] by human macrophages has been shown *in vitro* and was found to be mediated by NO.

To date, the exact role of CD23 during IgE-mediated immunity remains unclear. We recently showed that blocking CD23 by peptidic counter-structure abrogated IgE/antigen binding to human macrophages while the blocking of FcεRI had no effect on these cells [17]. In the present work, we used *T. gondii* to analyze the role of IgE during antiprotozoal activity of infected normal human macrophages. This opportunistic intracellular protozoan infects human macrophages and triggers Th1 and Th2 cytokines that enables host survival and long-term parasite persistence. Cytokine levels must be tightly balanced during this response, because their overproduction may cause immunopathology and host death [18], [19]. This phenomenon has been previously demonstrated in *T. gondii* infection of IL-10 knockout mice that succumb because of their inability to down-regulate parasite-induced proinflammatory cytokine production [20]. Th1-dependent IFN-γ production is a hallmark of effector immune response to acute infection with most intra-macrophagic parasites, including *T. gondii* [18], [21]. IFN-γ mediates the induction of the tryptophan-catabolising enzyme indoleamine 2,3-dioxygenase, as well as inducible nitric oxide synthase (iNOS), both known for their role in the defense against pathogens and inflammation [21]. Although NO was shown to be required for protection against intracellular *T. gondii*, the exact mechanism of iNOS promotion in human infected macrophages by IFN-γ is yet to be fully understood [22], [23]. Generation of NO through iNOS may also be achieved following cross-linking of surface FcεRII/CD23 in human and rat macrophages [8], [13]. Even if expressed by most macrophages, the role of CD23 as functional IgE receptor on these cells, together with its involvement during *T. gondii* infection remained unknown. Using infected normal human macrophages, we have analyzed the role of IgE and endogenous cytokines in the anti-parasitic activity of these cells. Our study has implications for the understanding of the biology of IgE during parasitic infection since it mediates CD23-cross-linking, NO generation, parasite killing and the down regulation of IL-10 levels produced by infected cells. These data corroborated the reported levels of cytokines during human infection and revealed a new mechanism of macrophage antimicrobial activity.

## Results

### IgE mediates intracellular elimination of parasites by human macrophages: Role of Fc*ε*RII/CD23

**Figure 1:**
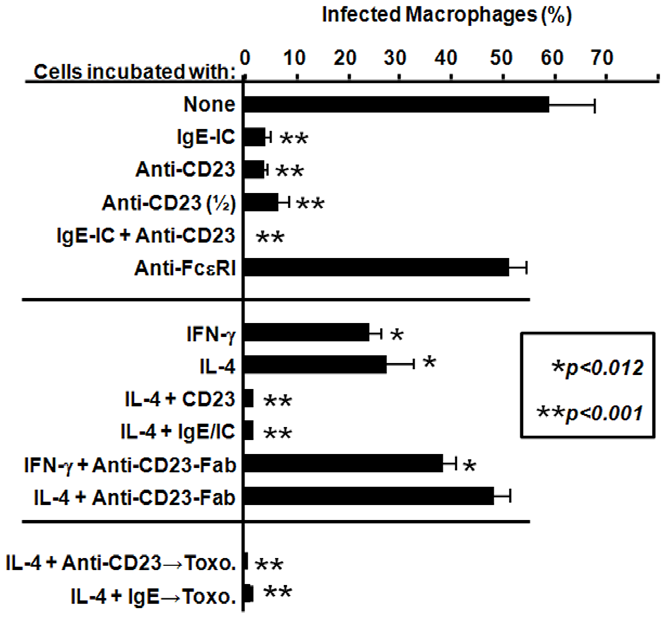
IgE-IC induces toxoplasmacidal activity of human macrophages through FcεRII/CD23 ligation. *T. gondii*-infected macrophages were incubated in the presence of IgE-IC, cross-linking anti-FcεRI (20 µg/ml), or anti-FcεRII (10, 20 µg/ml) McAb and the percentage of infected cells was assessed following 72 h incubation. Only IgE-IC and anti-FcεRII induced parasite elimination (upper panel). Infected cell incubation with recombinant IL-4 or IFN-γ induced parasite killing, reversed by the simultaneous blockade of CD23 cross-linking by Fab fragments of anti-CD23 McAb (20 µg/ml) (median panel). Cells were also treated 24 h with IL-4 and IgE-IC or anti-CD23 prior to cell infection. The lower panel shows that pretreatment of macrophages during 24 h with CD23 ligands enabled them to resist to *T. gondii* infection. Results show mean±SD from 3 distinct macrophage preparations, each done in duplicates. Asterisks show significance compared to infected cells cultured in medium alone.

Following contact with microbial antigens or their infection, macrophages acquired increased levels of surface CD23 [8], but the phenomenon was potentiated by IFN-γ or IL-4 at transcriptional level [24]. Following incubation with *T. gondii*, 54–72% of macrophages were infected and increased expression of CD23 was observed on their membrane (21–41% or 67–79% at 24 h and 48 post-infection respectively). Following 72 h incubation in medium alone, the percentage of infected cells was slightly modified (from 55% to 60%). *In vitro* death of a small percentage of infected cells could not be excluded. Addition of IgE-IC dramatically reduced the number of infected macrophages (p<0.0002, Figure 1, upper panel) and may be mimicked following the addition of cross-linking anti-CD23 McAb to these cultures (From 57% to 4% infected cells 72 h later). Combining both ligands resulted in a complete clearance of parasites (<2% infected cells remained), while cross-linking anti-FcεRI has no effect in this respect, supporting the role of CD23/FcεRII in IgE-mediated toxoplasmacidal activity. Of interest, pretreatment of infected cells with IFN-γ or IL-4, which increase CD23 surface expression in human macrophages, had additive effect to their parasiticidal activity (Figure 1). We also observed parasite killing with cells incubated with IFN-γ or IL-4 alone. This may likely be due, in part, to CD23 induction because simultaneous addition of anti-CD23-Fab fragments, which blocks FcεRII and prevents its cross-linking, significantly (p<0.004) reversed parasite clearance (Figure 1, middle panel). As simultaneous addition of IL-4 to CD23 ligands resulted in better elimination of *T. gondii* by human macrophages, we maintained these culture conditions for subsequent experiments. Finally, we asked if IgE-mediated activation could prevent macrophage infection with *T. gondii*. The cells were then treated 48 h with IgE-IC or anti-CD23 McAb before being incubated with parasites and the results clearly indicate that parasites fail to survive cell activation (Figure 1, lower panel). Microscopical analysis indicated that most parasites were killed following intracellular entry, although a small number of killed parasites were also detected on cell membrane (data not shown).

### Critical role of nitric oxide during IgE/IC-mediated *T. gondii* killing by human macrophages

**Figure 2:**
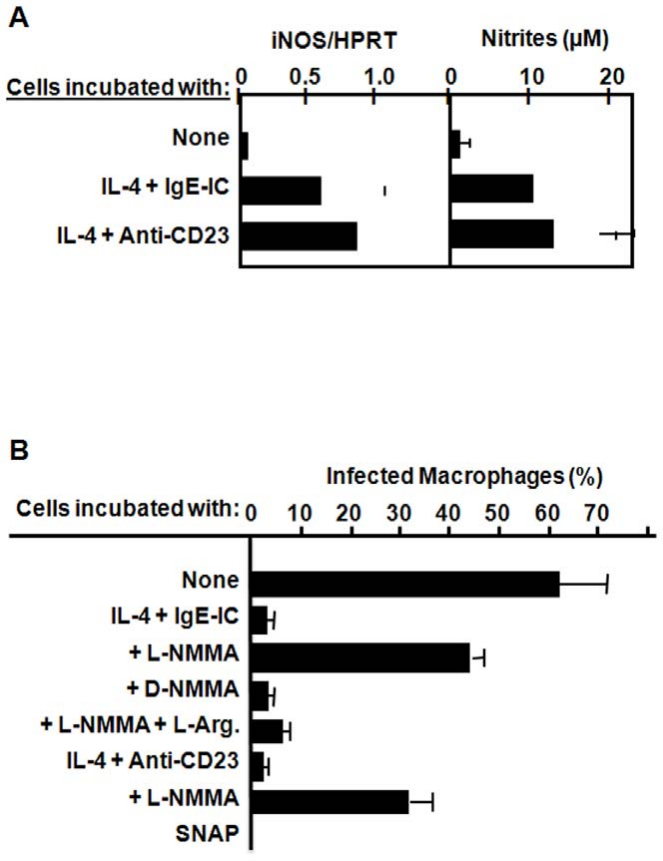
Involvement of iNOS pathway during IgE-IC-mediated activation of *T. gondii* killing by human macrophages. Infected cells were incubated in the presence of IL-4, IgE-IC or anti-CD23 McAb. (**A**) Cells were then collected (24 h) for iNOS-mRNA quantification and cell supernatants were harvested (72 h) to assess the levels of nitrites. Both conditions induced iNOS mRNA expression and consequent NO generation. (**B**) Cells were also incubated with an iNOS inhibitor (L-NMMA), a negative control (D-NMMA), or chemical NO-donor (SNAP). Following 72 h incubation, the percentage of infected macrophages was assessed. Addition of L-NMMA inhibited IgE-IC- or CD23-mediated parasiticidal activity (p<0.0008) and was reversed by L-arginine supplementation. Addition of chemical NO completely destroyed parasites without cell toxicity (>75% viable cells found in control cultures). Results show mean±SD for data from 3 distinct macrophage preparations, each done in duplicates.

The observed antimicrobial activity could be a direct consequence of CD23 engagement or, alternatively, could be induced by mediators generated by this pathway. Indeed, cross-linking of CD23 on human macrophages is known to mediate cell activation and the transcription of genes encoding various inflammatory cytokines including TNF-α, IL-1, IL-6 or IL-8 [25]. CD23 pathway also promotes the generation of superoxide, the transcription of iNOS gene and subsequent production of NO by human macrophages [8], eosinophils [26], and epithelial cells [27]. As NO mediates parasiticidal activity, we asked if CD23/NO pathway had a role in *T. gondii* killing by human macrophages. For this purpose, the amounts of nitrites, final metabolites of NO, were quantified in cell supernatants. As shown in Figure 2A, data indicate that FcεRII/CD23 stimulation induced the expression of iNOS mRNA. This finding was confirmed by the generation of nitrites in cell supernatants. Figure 2B further shows that the addition of the NOS inhibitor, N*^G^*-monomethyl-L-arginine (L-NMMA) significantly reversed parasiticidal activity (p<0.001) while D-NMMA had no effect. Addition of L-arginine to L-NMMA reversed NOS inhibition and restored parasite killing. CD23-mediated effect in inducing parasite killing may be mimicked by incubating infected cells with NO-releasing chemical, s-nitroso acethylpenicillamine (SNAP, Figure 2B). These data clearly support the role of NO during IgE-IC-mediated parasiticidal effect.

### TNF-α generation by CD23-stimulated human macrophages is not involved in *T. gondii* killing

**Figure 3:**
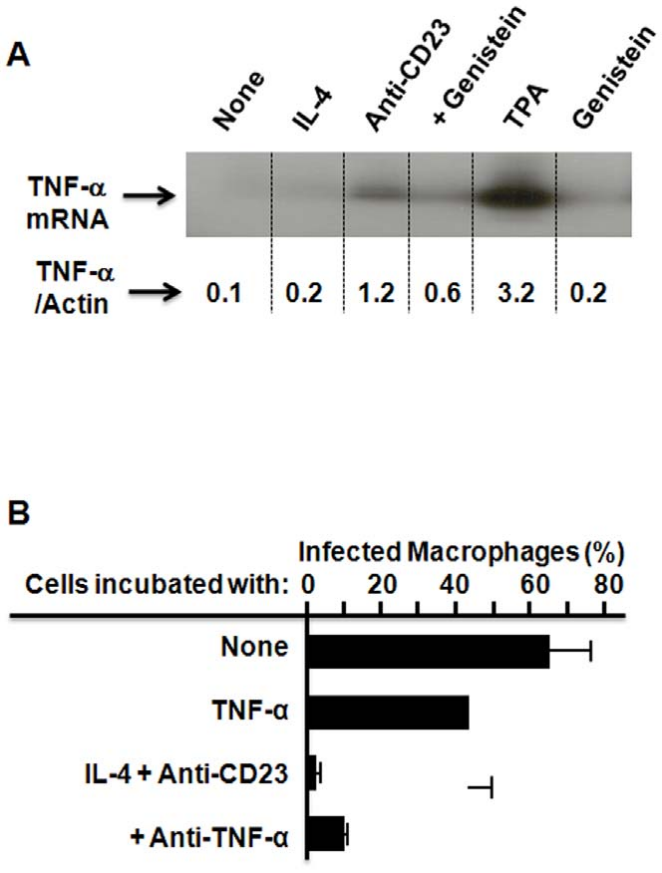
Minor role for CD23-induced TNF-α during activation of parasiticidal activity of human macrophages. (**A**) CD23 engagement induces TNF-α gene expression in human macrophages through tyrosine kinase-sensitive pathway. Data from one donor was shown, out of two. (**B**) Recombinant TNF-α (100 IU/ml) induced low but significant elimination (p<0.007) of parasites in infected cells while anti-TNF-α McAb (20 µg/ml) had limited effect on CD23-mediated parasiticidal activity of human macrophages. Results show mean±SD from three distinct MDM preparations following 72 h incubation, each done in duplicates.

We then investigated the role of TNF-α during IgE-IC-mediated *T. gondii* killing because this cytokine has been suggested as an toxoplasmacidal mediator [18], [19]. Following CD23 activation, significant increase in TNF-α mRNA level was observed in CD23-activated cells, compared to controls (Figure 3A). Moreover, we observed reversion of this phenomenon by genistein, an inhibitor of protein tyrosine kinase (PTK) activity [28], a critical intracellular signal transduction pathway during CD23-mediated cell activation [29]. CD23 also led to increased production of TNF-α in cell supernatants (from 42±24 to 425±134 pg/ml, p<0.0003, mean±SD of four distinct MDM preparations). In non-activated macrophages, exogenous recombinant TNF-α induced low but significant parasite killing (p<0.008, Figure 3B), while simultaneous addition of neutralizing anti-TNF-α antibody during CD23 activation did not significantly reduce parasite clearance (p = 0.09). This result suggested little or no involvement of this cytokine during CD23 engagement.

### IL-10 decreased IgE-mediated *T. gondii* killing by human macrophage

**Figure 4:**
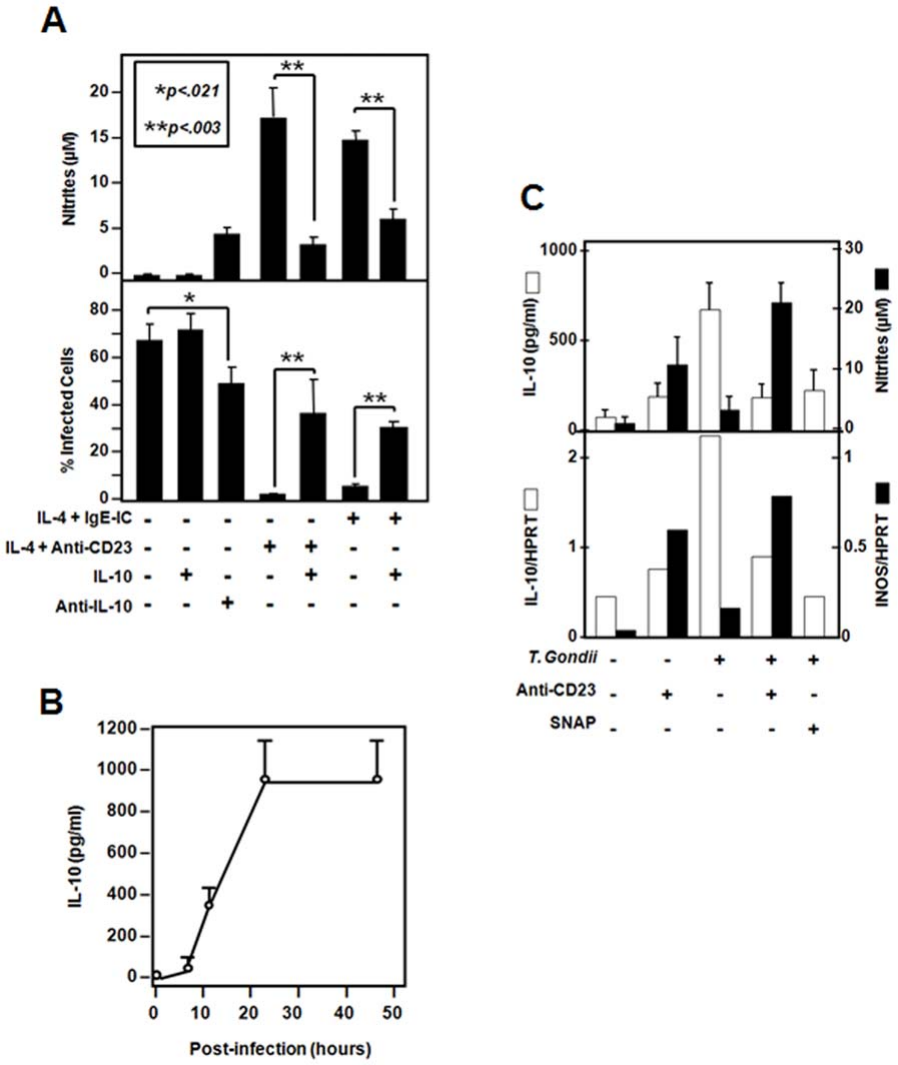
Inhibition of IgE-IC-mediated toxoplasmacidal activity of human macrophages by endogenous and exogenous IL-10. (**A**) Simultaneous addition of recombinant IL-10 (10 ng/ml) to CD23-engaged macrophages significantly decreased their ability to eliminate *T. gondii*. Addition of neutralizing anti-IL-10 (20 µg/ml) McAb increased cell resistance to infection in the absence of CD23 engagement. Results show mean±SD from 3 distinct MDM preparations following 72 h incubation, each done in duplicates. (**B**) Infection with *T. gondii* induces IL-10 generation by human macrophages. Results show mean±SD from two distinct MDM preparations, each done in duplicates. (**C**) Infected or uninfected cells were incubated with anti-CD23 or chemical NO (SNAP) and were collected 24 h later for iNOS- and IL-10-mRNA quantification and cell supernatants were harvested (72 h) to assess the levels of nitrites and IL-10. CD23 engagement reduced *T. gondii*-mediated IL-10 increase at both mRNA and protein levels and was inversely correlated with iNOS expression by infected human macrophages. Chemical NO (SNAP) also reduced the IL-10 expression from infected macrophages. Results show mRNA quantification from one representative macrophage preparation, out of two and mean±SD from 2 distinct MDM preparations following 72 h incubation for the quantification of mediators, each done in duplicates.

The regulation of toxoplasmacidal effects of CD23 pathway was investigated. We have previously shown that IL-10, a Th2 cytokine produced during various inflammatory responses, down-regulated CD23/NO pathway in human cells [10], [24], [30]. In addition, *T. gondii* infection was previously shown to induce IL-10 production *in vivo* and this cytokine may therefore moderate the inflammatory response of macrophages [31]. We here show that the addition of recombinant IL-10 during CD23 engagement reduced parasite killing and NO generation from infected human macrophages (Figure 4A). Of interest, addition of neutralizing anti-IL-10 to macrophages resulted in a significant reduction of infected cell number and increased NO generation by these cells, even in the absence of exogenous stimulation (Figure 4A). This suggested that autologous IL-10 may be induced following cell infection. To confirm this observation, we quantified IL-10 levels in MDM supernatants following their infection with *T. gondii*. A time-dependent increase of IL-10 levels in human MDM supernatants was observed (Figure 4B). Data from Figure 4C further indicates that *T. gondii* induced IL-10 expression at both mRNA and protein levels and that IL-10 expression inversely correlated with iNOS-mRNA and NO levels in MDM. Of particular interest, while CD23-mediated activation of uninfected macrophages induced low IL-10 levels (Figure 4C), CD23 stimulation or chemical NO decreased IL-10 expression in human *T.gondii*-infected macrophages at both RNA and protein levels.

### *In vivo* expression of IL-10, NO derivatives and IgE in patients' sera

**Figure 5:**
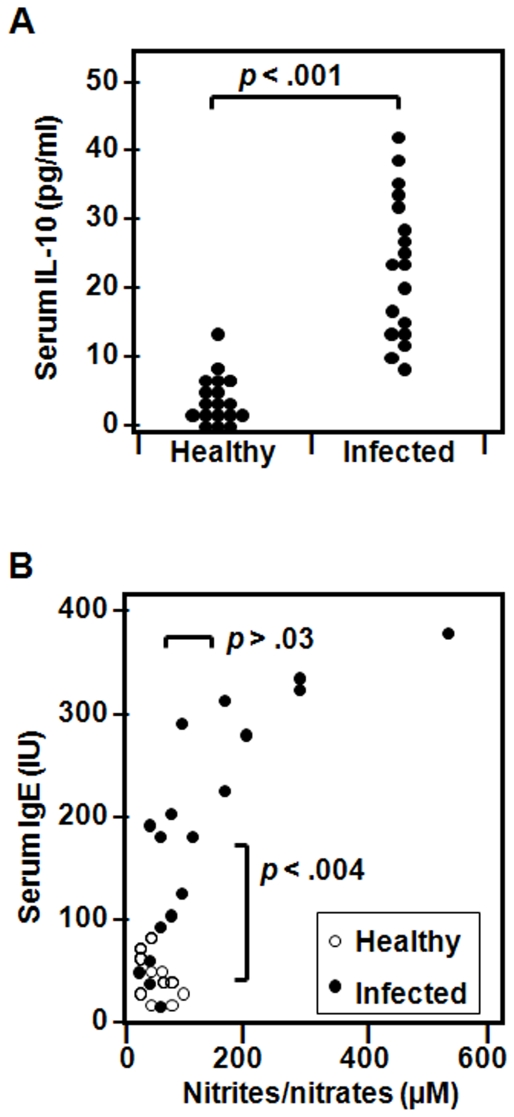
The presence of IL-10, IgE and nitrites in sera from patients infected with *T. gondii*. Freshly isolated **s**era from 18 *T. gondii* infected patients or 11–18 uninfected controls were tested for the levels of IL-10, IgE and nitrites. (**A**) Infected patients have significantly high serum levels of IL-10 compared to controls. (**B**) Elevated IgE levels were detected in infected patients compared to controls, while concentrations of serum nitrites were close to normal values except to 6/8 patients with high IgE (>200 IU) levels (p<0.018).

*In vitro* data lead us to investigate the *in vivo* presence of various above inflammatory mediators described in sera from *T. gondii*-infected patients compared to non infected controls. Sera from infected patients and uninfected donors were tested for their IL-10, IgE and nitrites/nitrates content. Data in Figure 5A clearly shows significantly higher levels of IL-10 in sera from infected symptomatic patients versus uninfected controls. Figure 5B also indicates that sera from *T. gondii*-infected donors contained significantly elevated IgE levels compared to controls (p<0.004), while the levels of nitrites were variable and mostly closed to those from uninfected controls.

## Discussion

Immune response to parasite infection is often correlated with an increased expression of IgE in mammals, which is believed to play a protective role against worms [1]. Meanwhile, the role of IgE and its receptors during antiprotozoal immune response still remains to be fully understood. Our study clearly shows that macrophages, in the absence of FcεRI, express FcεRII at enough surface levels that enable them to cross-link these receptors by IgE-IC or other physiologic ligands. Consistently, CD23 expression following infection with *T. gondii* parasites may be due to their ability to induce the transcription factor STAT6 [11], which is involved in the induction of Th-2 gene promotion including CD40, CD23 and IgE. Furthermore, maternal immunity to *T. gondii* has been associated with specific IgE detection in cord blood [32]. Parasite antigens may also activate p38 mitogen-activated protein kinase P [33], which has been reported to mediate IL-4-induced expression of CD23 [29]. Cytokines from infected or neighboring cells are important in macrophage activation, CD23 expression and the generation of parasiticidal agents. Therefore, the balance of *in situ* cytokines such as TNF-α, IL-12, IL-10, IL-4 and IFN-γ has the dual effect of keeping the host alive and/or inducing inflammatory disease when overproduced. Our data suggest that following *T. gondii* infection, macrophages acquire simultaneously CD23 signaling and the availability of IgE-IC, their natural ligands. Previous studies conducted in various parasitic and microbial infections have shown CD23 expression together with its role in intracellular and extracellular killing of pathogens by macrophages [3], [8], [10]. Cell activation via CD23 requires the presence of appropriate physiologic ligands [25] such as immunoglobulin E, CD21, CD11b/c, CD47-vitronectin, and mannose-containing proteins [7]. In our study, CD23-crosslinking by IgE/anti-IgE or anti-CD23-McAb was shown to mediate parasite killing in human macrophages. This finding supports the role of CD23 as a functional IgE receptor in these cells. This was further confirmed through the ability of Fab fragments of anti-CD23 McAb, which bind CD23 without cross-linkage, to block cell activation and reversed the antimicrobial effect. CD23 pathway also account for IFN-γ- and IL-4-mediated parasiticidal activities as both cytokines were found to be less efficient following CD23 blockade with anti-CD23 Fab fragments. Moreover, both cytokines were well known for their ability to increase surface CD23 expression on human macrophages. Therefore, the acquisition of surface CD23 and the availability of its physiological ligands in cell environment may argue for its important role in human immune response to *T. gondii* infection.

CD23-mediated NO generation seems to play a major role during parasiticidal activity of macrophages. NO. is a highly reactive and diffusible free radical, soluble in both lipids and water. It reacts with oxygen and reactive oxygen intermediates forming NO_2_, NO_2_^−^, NO_3_^−^, N_2_O_3_, and the highly parasiticidal ONOO^−^ [34]. Inducible nitric oxide synthase is the major generator of NO in MDM and is a tightly regulated enzyme. Therefore, CD23 engagement, which mediates increased transcription of iNOS in human macrophages, induces NO generation following *T. gondii* infection. Furthermore, in murine macrophages, parasites may escape this pathway through their ability to decrease iNOS expression by infected cells via an unknown mechanism [35]. One plausible hypothesis may be related to their ability to generate Toll-like receptor-induced arginase 1 in macrophages which may act in reducing the intracellular arginine level, the latter being necessary for an optimal iNOS function [35]. Indeed, dominated T helper type 2 responses in parasitic disease increased arginase expression by IL-4 and IL-13 signaling through the transcription factor STAT6 [36].

Among cytokines, TNF-α plays a critical role in host defense against intracellular parasite infections [18]. CD23 engagement is responsible for a significant increase in TNF-α secretion by MDM. However, the addition of anti-TNF-α during CD23 activation has little or no impact on the microbiocidal activity, suggesting a minor role for this cytokine in this system. In this context, it is striking to note that TNF-α was previously shown to be necessary for an optimal NO production from human infected macrophages [8], [37]. By contrast, CD23 pathway differs from Toll-like receptor mediated toxoplasmacidal activity, where a critical role for TNF-α [38] while NO seems to play a marginal role [39].

Like other inflammatory mediators, IL-10 is produced following CD23-mediated activation of normal uninfected macrophages [17], [30]. The levels of IL-10 inversely correlate with the generation of other inflammatory mediators, including NO [10], [24], [30]. These data corroborate studies conducted in IL-10^−/−^ mice that have observed lethal or exacerbated inflammatory disease following *Toxoplasma* infection [20]. Accordingly, *in vitro* infection of human macrophage with *T. gondii* resulted in an increased expression of IL-10 from these cells, which inversely correlated with iNOS expression and NO generation. In contrast to uninfected cells, CD23-activation of *T. gondii*-infected macrophages had no additive effect on IL-10 production but rather down regulated this cytokine (Figure 4C). This clearly evidenced *T. gondii* strategy to escape human immune response as incubation of infected macrophages with neutralizing anti-IL-10 antibody led to increased elimination of parasites without additional *in vitro* activation. These *in vitro* data corroborate *in vivo* observations as we clearly ascertained that acute *T. gondii* infected patients had increased IgE and IL-10 levels in their sera, while NO levels were close to normal controls. Imunoregulation by *T. gondii* infection also prevents allergic immune reponses and IgE production in mice [40]. Together, our findings clearly demonstrate the role of IgE-IC in mediating intracellular protozoal elimination through their ability to ligate membrane bound CD23/FcεRII. Moreover, the latter evidence supports the mechanism leading to the frequently reported increase of peripheral IgE levels that occur in the course of these infections. IgE-dependent immune response and protozoal elimination also raise the question of the possible interaction with nematode infections and disease outcome [41], [42]. Finally, high levels of TNF-α and NO mediated through IgE generation may also account for deleterious chronic inflammatory diseases that are observed during many parasitic infections.

## Materials and Methods

### Ethics statement

Informed written consent was given by the blood volunteer donors and the study was approved and strictly followed the ethics guidelines of Medical Ethical Committees at the University of Pierre et Marie Curie, Paris, France, and conducted under full compliance with government policies and the Helsinki Declaration. Patients' data were obtained from routine investigations or provided from blood samples beyond the procedures aimed for diagnosis or management of patients and strictly followed the ethics guidelines of local Medical Ethical Committees (see above).

### Parasite cultures

*Toxoplasma gondii (T. gondii)*. Virulent RH strain was maintained in Balb/c mice (Iffa-Credo, St German/Arbresle, France) by intraperitoneal passage and isolated by peritoneal lavage following filtration. Tachyzoites were used in our study and their viability controlled by trypan blue dye exclusion and only parasite preparations with >95% viable cells were used. All animal procedures were performed in strict accordance with the guidelines issued by the European Economic Community “86/609” and local authority approval (Prefecture de la Gironde N° 33/03239).

### Human cells and infection

Peripheral blood samples pre-tested for the absence of HIV or hepatitis B and C virus infection were obtained from healthy volunteers (Blood Transfusion Center, Pitié-Salpêtrière Hospital, Paris, France). Peripheral blood-derived mononuclear leukocytes were obtained by Ficoll gradient separation and suspended in McCoy 5A modified culture media supplemented with 100 U/ml penicillin, 100 µg/ml streptomycin, 25 mM HEPES, 0.1 mM 2-mercaptoethanol, 2 mM sodium pyruvate, 0.2 mM L-cysteine, 5 µg/ml polymyxin B and 10% fetal calf serum (FCS) (all from Invitrogen, Paisley, UK). All the above culture medium, chemicals, and FCS were endotoxin-free and tested for the absence of direct activation effect on human monocytes (CD23 expression and TNF-α production as activation markers). Monocytes were subsequently separated from other leukocytes by adherence to FCS-coated culture flasks and re-incubated for additional five days in the same culture conditions. Following these procedures, >95% of cells expressed CD14 antigen and displayed cytochemical characteristics of monocyte-derived macrophages (MDM) [8]. Cells were collected and re-incubated (2,5×10^6^/ml) in Lab-tek culture “chambers” during 60 min in the presence of *T. gondii* tachyzoites at 1∶2 cell∶parasites ratio in DMEM complete medium at 37°C in humidified air, containing 5% CO_2_. They were washed and re-incubated for additional 24 h at 2×10^5^ MDM/ml before being treated with various molecules. This led to the infection of 57–73% MDM as counted by direct microscopical analysis of May-Grunwald Giemsa (MGG)-stained cytospin preparations. For each culture condition, 500 MDM were examined in two separate experiments. To measure MDM viability and apoptosis, externalization of membrane phosphatidylserine was analyzed using annexin V-FITC and propidium iodide kit (Immunotech, Marseille, France). The percentage of apoptotic/infected cells was obtained following count of 200 cells in each well under fluorescence microscopy. Concurrently, serum levels of IgE, nitrites and IL-10 were analyzed from samples collected beyond routine exploration of HIV-free patients with symptomatic primary *T. gondii* infection. Values were compared to those found in sera from normal healthy *T. gondii* infection-free donors.

### Human cell activation

Cell surface expression of CD23 and CD14 was measured by flow cytometry, using FITC-labeled McAbs (Beckman-Coulter, Fullerton, CA, USA). Infected MDM were directly activated through CD23 pathway while CD23 cell surface expression was induced in uninfected MDM in the presence of recombinant human IFN-γ (100 IU/ml, Clinisciences, Montrouge, France) or IL-4 (10 ng/ml, gift from Novartis, Basel, Switzerland) as described elsewhere [8], [24]. CD23-positive cells were incubated in the presence of human IgE/anti-IgE (10 µg/ml of each, Nordic, Tilburg, Netherlands) immune complexes or cross-linking anti-CD23 monoclonal antibody (CD23-MAb, clone 135, IgG1κ, 20 µg/ml, Novartis, Basel, Switzerland) or anti-FcεRI (clone 15-1) as detailed elsewhere [9]. MDM cultures were supplemented with the chemical NO donor, SNAP (S-Nitroso-N-acetylpenicillamine; Coger, Paris, France), recombinant human IL-10 or TNF-α, neutralizing anti-human-TNF-α (Genzyme, Cambridge, MA, USA), neutralizing anti-human IL-10 McAb, isotype-matched control for anti-CD23 (anti-CD8, R&D Systems, Abington, UK), or genistein (Sigma-Aldrich, St Quantin Fallavier, France).

### RNA preparations and detection

Following cultures, total cell RNA was extracted using RNeasy kit (Qiagen, Hilden, Germany). Reverse transcription-PCR was performed with an automatic thermal cycler (iCycler, Biorad) using the following specific primers: iNOS mRNA sense (5′-ATGCCAGATGGCAGCATCAGA-3′, exon 8) and iNOS mRNA antisense (5′-ACTTCCTCCAGGATGTTGTA-3′, exon 11). IL-10 mRNA sense, 5′-GCAACCTGCCTAACATGCTTCG-3′; IL-10 mRNA antisense, 5′-GAAGATGTCAAACTCACTCA TGGC-3′ (exon 11). Hypoxanthine phosphoribosyltransferase (HPRT) mRNA sense (5′TATGGACAGGACTGAACGTCTTGC-3′) and HPRT mRNA antisense (5′-GACACAAACATGATTCAAATCCCTGA-3′) primers were used as controls. The iNOS messenger is represented by a 371-bp band, IL-10 by 388-bp band, whereas a 496-bp band indicates the HPRT messenger. Signal intensity was compared to HPRT control AlphaImager HP automatic image capture software (Alpha-Innotec, San Leandro, CA, USA). Ten µg of total RNA was analyzed by Northern blot hybridization as described for the expression of TNF-α mRNA using 32P-labeled probe [43], a 685-bp *Bg*I-*Pst* I fragment of pE4 (American Type Culture Collection, Rockville, MD, USA), compared to a 1.1-kb *Pst* **I** βActin (a gift from D. Stehelin, Institut Pasteur, Lille, France).

### Analysis of NO, IgE and cytokine quantification

Culture supernatants (48–72 h) or sera were tested for the stable end-product of NO, NO_2_^−^ using the Griess reaction modified as detailed elsewhere [44]. This method gave a sensitivity limit of 0.2 µM if low NO_2_^−^ medium (DMEM) was used. TNF-α and IL-10 (Bender Medsystems, Vienna, Austria) and total human IgE (Mabtech AB, Sophia Antipolis, France) were quantified using specific ELISA kits.

### Statistical analysis

Comparisons of data from infected patients were assessed using Fischer's exact test for proportions and Mann-Whitney U test for quantitative values. Results from *in vitro* cultures were analyzed and compared using the Student *t*-test for paired data. *P*<0.05 was considered to be significant.
